# Cancer diagnostics based on plasma protein biomarkers: hard times but great expectations

**DOI:** 10.1002/1878-0261.12809

**Published:** 2020-11-17

**Authors:** Ulf Landegren, Maria Hammond

**Affiliations:** ^1^ Department of Immunology, Genetics and Pathology Uppsala University and SciLifeLab Uppsala Sweden

**Keywords:** affinity‐based protein assays, blood markers, liquid biopsy, protein biomarkers, proximity ligation assay, tissue‐specific expression

## Abstract

Cancer diagnostics based on the detection of protein biomarkers in blood has promising potential for early detection and continuous monitoring of disease. However, the currently available protein biomarkers and assay formats largely fail to live up to expectations, mainly due to insufficient diagnostic specificity. Here, we discuss what kinds of plasma proteins might prove useful as biomarkers of malignant processes in specific organs. We consider the need to search for biomarkers deep down in the lowest reaches of the proteome, below current detection levels. In this regard, we comment on the poor molecular detection sensitivity of current protein assays compared to nucleic acid detection reactions, and we discuss requirements for achieving detection of vanishingly small amounts of proteins, to ensure detection of early stages of malignant growth through liquid biopsy.

## Graphical abstract

Liquid biopsy via protein biomarkers holds out hopes for early detection of malignancies and monitoring disease course. However, despite extensive efforts current protein assays largely fail to provide the required diagnostic accuracy. In this Review we propose that realizing the hopes for valuable diagnostic assays will require a combination of intelligent selection of biomarker candidate proteins, and substantially improved assay techniques.

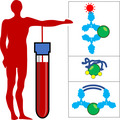

**Abbreviations**
ACPP: acid phosphatase
AFP: alpha‐fetoprotein
CEA: carcinoembryonic antigen
CTLA4: cytotoxic T lymphocyte‐associated protein 4
FDA: (US) Food and Drug Administration
GTEx project: Genotype‐Tissue Expression project
HCA: human cell Atlas
IL‐6: interleukin 6
PD‐1: programmed cell death 1 receptor
PD‐L1: programmed cell death ligand 1
PSA: prostate‐specific antigen
TCGA: The Cancer Genome Atlas

## 1 Introduction

**Fig. 1:**
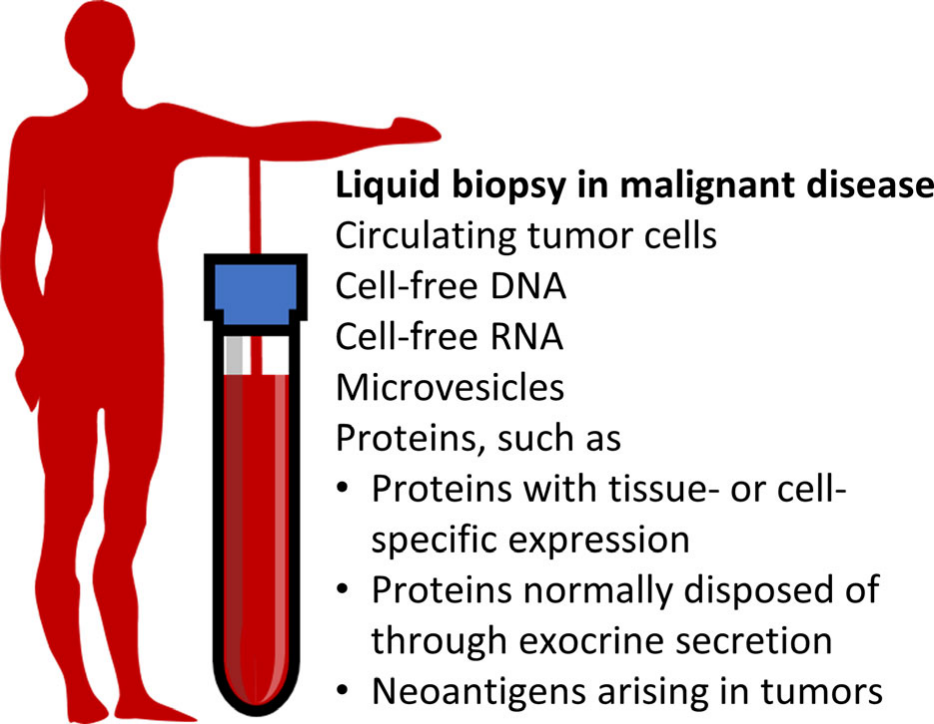
Liquid biopsy provides opportunities to diagnose and monitor malignancies anywhere in the body by sampling blood or other body fluids. The figure lists some common targets of liquid biopsy, including relevant categories of proteins.

Liquid biopsy offers the possibility to assess the state of health of specific tissues in the body by detecting their released molecules in easily accessible body fluids such as blood. The approach offers a means to screen for disease processes anywhere in the body, and it represents a simple and practical alternative to taking biopsies of specific organs for molecular analysis. For example, tumor cells disseminated in the blood stream can serve as a measure of tumor load and risk of metastasis, and they also offer an opportunity to investigate detailed molecular characteristics of a tumor. Besides cells, also individual molecules released in blood can carry crucial information about tissue health, and both DNA and RNA molecules are useful targets for liquid biopsy in cancer and other diseases (Fig. 1).

Proteins found in blood plasma have a long, if somewhat troubled, history as biomarkers of cancer, one that predates the several more recently introduced classes of biomarkers for liquid biopsy. A very broad literature exists that describes the search for protein marker, but we will make no attempt to review this here. Suffice it to say that clinically useful protein markers have been hard to find, and the acceptance of new diagnostic protein biomarkers for cancer in blood samples has proceeded at a very slow pace, despite the rapid increase in general molecular insights and technological progress for molecular detection reactions. Some improved selections of biomarkers are noted; for example, acid phosphatase (ACPP) has been replaced as a marker for prostate cancer by the more specific, but still not quite satisfactory, prostate‐specific antigen (PSA) [1], which is associated with frequent false positives in cancer diagnostics. A slowly expanding range of other protein markers is commonly used to detect malignancy, including AFP (alpha‐fetoprotein), CA‐125, CEA (carcinoembryonic antigen), CA15‐3, CA19‐9 [2, 3, 4], and a few more, but their clinical utility is limited, even when they are applied in combination (for a current list of FDA (US Food and Drug Administration)‐approved protein biomarkers, see table S1 of Suhre *et al*., 2020 [5]). Accordingly, protein liquid biopsy at present remains far from satisfactory, although combinations of protein and DNA biomarkers may improve prospects [6].

It is reasonable at this point to ask whether we have found all useful protein biomarkers, and if current protein assay techniques are able to meet the requirements for liquid biopsy in cancer and other diseases. The purpose of this brief perspective is to argue that we have not been looking in all the right places for suitable protein biomarkers, and the vision provided by current assay techniques may have been too poor to find and evaluate the really good markers—but that there now is hope that all this may be about to change.

## 2 Potential protein biomarkers of malignant disease

So, where should one look for promising protein markers for liquid biopsy? Many current plasma protein biomarkers, such as proteins related to the coagulation system or cytokines, provide highly relevant information about system‐wide states of health, but they do not point to any particular organ as the locus for a disease process, and they can therefore not be considered as markers for liquid biopsy. Recently available techniques to screen for large sets of plasma proteins are promising for revealing characteristic changes of plasma proteins in a large variety of disease states [7, 8, 9], but more directed strategies may be needed to improve chances to uncover markers that are useful for early detection or highly sensitive follow‐up of cancer patients. For this purpose, we need to ask how malignancies and other tissue‐specific dysfunctions could lead to release of increased levels of tissue‐specific proteins in blood (Fig. 1)? Below we discuss types of proteins that may be released from tissues to the circulation through disease‐specific processes.

### 2.1 A questionable role for oncofetal antigens

The idea that cancers may reinitiate expression of proteins normally only present in embryogenesis, thereby providing markers for tumors somewhere in the body in adult life [10], has not proven very helpful. CEA or carcinoembryonal antigen is an example of a protein that has been thought to have this characteristic [11], but as a biomarker it is of only limited help in diagnosis of malignancy via blood sampling [12]. Examples of other proteins, reportedly belonging in the same category, are glypican 3 [13] and insulin‐like growth factor II mRNA‐binding protein [14], but neither of these two proteins have been established as plasma cancer biomarkers. Accordingly, it remains doubtful whether useful protein biomarkers can be found that are predominantly or exclusively expressed in embryogenesis and also in malignancy.

### 2.2 Proteins with tissue‐ or cell‐specific expression

It is perhaps more reasonable to pin one's hope on proteins with limited expression across tissues as possible markers of the health of those particular tissues [15, 16]. PSA and ACPP are examples of such tissue enriched biomarker proteins, and many more are being explored [17, 18]. Besides selective gene expression, tissue‐specific protein expression may also result from mechanisms such as differential splicing, giving rise to tissue‐specific variants. Proteins can furthermore vary due to posttranslational processing and modifications. It has been estimated that in total millions of proteoforms can exist in the human body [19]. One example of a protein modification used as a biomarker for malignant disease is CA19‐9 [20], where the targeted epitope is a glycosylation, Sialyl‐Lewis^A^ [21].

Information about specific protein expression throughout the body currently grows by leaps and bounds. Over several years, mRNA sequencing of specific tissues has provided comprehensive views of gene expression, indicating what proteins can be expected to be present in the different organs of the body. These investigations also delineate which of those proteins might be expressed in single tissues, or at least limited groups of tissues. The Human Protein Atlas (https://www.proteinatlas.org) [22] represents a useful repository of information about mRNA and protein expression across multiple healthy tissues and the most common cancers, while the Genotype‐Tissue Expression (GTEx) project (https://gtexportal.org) [23] annotates tissue‐specific gene expression in relation to genetic variation in close to a thousand healthy individuals.

However, organs and tissues typically represent complex assemblies of distinct cooperating cells, cells that may differ widely in ontogeny, function, and in gene expression repertoires. Increasing amounts of information about RNA expression, and by inference or direct examination also proteins, is now becoming available for the individual cells that make up tissues and organs. In this regard, we are at long last living up to the aims of Rudolf Virchow, who in 1858 expounded the need to establish a true cellular pathology [24]. The Human Cell Atlas (HCA, https://www.humancellatlas.org) project is a grand worldwide effort to characterize the molecular composition of all cells in the human body that differ in ontogeny and state [25]. Systematic sequencing of transcriptomes of individual cells in bodily organs within HCA and other research contexts [26] is now building an invaluable resource of information about tissue‐specific proteins. Elevated levels of such proteins in blood and other body fluids may point to disease processes that have the effect of disseminating proteins from those tissues.

### 2.3 Selective dissemination of proteins to blood

While tissue‐specific proteins hold out hopes as promising biomarkers, it is not obvious how such proteins would stand out in plasma samples from patients with malignant disease. In other words, why would a malignancy, preferably still small, localized and at a stage where curative extirpation might be possible, lead to the release of tissue‐specific proteins in blood at levels that exceed those emanating from a much greater mass of healthy tissue in the same organ?

Tumor‐specific elevated release of proteins may result from the fact that tumors tend to undergo more frequent necrotic events, because of uncontrolled growth that is poorly matched to vascularization [27]. It is reasonable to expect that necrotic tissues might release disproportionate amounts of proteins into the bloodstream [28].There could also be other reasons cancers might cause release of protein to the blood stream, having to do with the properties of the cells they originate from, as outlined below.

Most cancer deaths are due to carcinomas, that is, cancers that arise in epithelial tissues [29]. This is true for the most common tumors of the lung, breast, prostate, colon, etc. (https://www.cancer.org/research/cancer‐facts‐statistics.html#). Histologically, the cells of origin of these cancers are localized on a basal membrane on inner or outer surfaces of the body. Once these tumors grow beyond the *in situ* stage, they no longer respect the basal membrane as a demarcation zone, and their protein cargo may more readily reach the circulation than proteins expressed in healthy tissue. Moreover, epithelial cells can be engaged in exocrine secretion, delivering specific proteins into the lumen that they are lining [30]. Disordered growth of such cells may prevent proteins and other components that are secreted from tumor cells or by their crowded normal neighbors from reaching their normal destination. A well‐known example is the jaundice seen in advanced cancer of the pancreas when bilirubin fails to be disposed of through normal channels [31]. Similar mechanisms could potentially lead to increased blood levels of secreted proteins. A prominent case in point is the already mentioned prostate cancer marker protein PSA, which in a healthy man is present at an approximately million‐fold lower concentration in plasma compared to seminal fluid. Indeed, as is well‐known PSA is regularly present at elevated plasma levels in prostate cancer, but this can also be true in benign conditions of the prostate such as inflammation and hyperplasia, reducing the utility of this cancer marker [17].

In conclusion to this section, malignancies can manifest through increased release of tissue‐specific proteins to blood and other body fluids via a number of mechanisms. Accordingly, such proteins represent prime candidates for protein biomarkers in liquid biopsy.

### 2.4 Protein constellations on microvesicles

Besides individual proteins, epithelial cells may also secrete exosomes and other membrane‐coated microvesicles that may serve as biomarkers [32, 33]. For example, so‐called prostasomes can prove useful as cancer markers [34]. These are microvesicles that like PSA are actively secreted by the prostate in high numbers to seminal fluid, and that only occur at far lower concentrations in plasma from healthy men. In general, individual surface proteins or combinations thereof on microvesicles may provide interesting tissue‐specific markers for liquid biopsy. We have recently described a technique to map constellations of protein markers on large sets of individual microvesicles as a means to identify potentially diagnostic microvesicles [35]. In this context, it is pertinent to point out that some of the proteins detected in protein assays of plasma samples may well be located on the surfaces of microvesicles.

### 2.5 Proteins mutated in tumors as potential blood biomarkers

While oncofetal antigens may be a lost hope as a source of tumor‐specific markers, it is becoming increasingly clear that tumors regularly exhibit tumor‐specifically modified proteins or neoantigens, arising as a consequence of mutations. These neoantigens attract interest as they may evoke host responses to combat the tumors, but they may also serve as targets for diagnostic or therapeutic applications [36, 37]. Recent dramatic successes with immune oncological therapies targeting proteins like PD‐1 (programmed cell death 1 receptor), PD‐L1 (programmed cell death ligand 1), and CTLA4 (cytotoxic T lymphocyte‐associated protein 4) [38] indicate that many tumors in fact do provoke specific immune reactions. Those tumors that progress may have found ways to neutralize such defenses in a manner that can sometimes be overcome by the novel therapies. The targets of putative specific immune reactions in malignancy have rarely been characterized in any molecular detail, but it is plausible that novel epitopes exposed on mutated proteins in tumors constitute new proteoforms that may break tolerance and initiate specific T and/or B cell responses [39, 40]. Reading frameshifting insertions or deletions in coding regions and mutated stop codons can all give rise to new peptide sequences. Since there are only three stop codons out of the 64 codons, translation of the altered codons beyond such a mutated site can produce novel, potentially antigenic stretches of up to a few tens of amino acids before a new stop codon is reached by chance. Also, more subtle changes due to missense mutations might be recognized as foreign to the body and trigger immune reactions [41, 42, 43]. All in all, both missense mutations and larger frameshift mutations could yield tumor‐specific target proteins for liquid biopsy.

### 2.6 Nonrecurrent vs. recurrent mutations

Specific mutations in suppressor genes that alter the function of proteins normally preventing neoplastic growth typically differ among patients, since any number of changes may achieve the purpose of knocking out the function of the gene products. Mutations that arise in innocent bystander proteins, mutated as a consequence of a tumor‐specific inability to repair genetic mishaps, are even more likely to be unique for individual patients. In order to target such patient‐specific mutant proteins for diagnostic purposes, specific reagents and assays would have to be established for individual patients. It is relatively straightforward to device regents to detect patient‐specific mutations in circulating tumor DNA for following the course of disease using methods such as digital PCR or DNA sequencing. A corresponding approach for analyzing patient‐specific mutated proteins is presently impractical, however, because of the substantially greater difficulty of generating specific affinity reagents for proteins rather than for DNA.

A more promising avenue could be to target the smaller but nonetheless considerable number of recurring mutations that serve to activate products of dominant oncogenes such as for example the RAS genes, and in particular KRAS [44]. Only a limited subset of mutations has the potential to lock proteins in an on‐state that promotes tumor growth [45], and accordingly, these same mutations can be seen in tumors from many different patients. Public repositories of cancer genomes, such as The Cancer Genome Atlas (TCGA, https://www.genome.gov/Funded‐Programs‐Projects/Cancer‐Genome‐Atlas), enable systematic studies of such recurrent cancer mutations. Antibodies are already available against some recurrent mutations [46, 47], and this range could be expanded. Given sufficiently selective and sensitive protein assays, a repertoire of tests targeting recurrent mutant forms of dominant oncogenes could therefore furnish generally useful protein assays for follow‐up and maybe also for *de novo* detection of malignant disease, complementing analyses for tumor‐specific mutations at the DNA or RNA level. It is not yet possible to say if protein assays could present advantages over DNA analyses, for example due to the potentially greater numbers of mutant proteins for each mutant DNA or RNA copy, higher stability or more efficient release to, for example, blood.

Rounding off this section, we conclude that an intelligent choice of biomarker candidates with promising expression characteristics or tumor‐selective properties may increase chances to identify valuable protein biomarker candidates for liquid biopsy. Analyses of such markers will pose stringent requirements for the detection techniques applied. For example, parallel analyses of multiple biomarker proteins emanating from the same tissue and thus reinforcing each other can strengthen diagnostic accuracy by reducing risks of spurious results [2, 48, 49, 50]. Analysis of leakage markers from tumor tissue may also require greatly improved assay sensitivity as outlined below.

## 3 Sensitive and selective protein assays for liquid biopsy

A fundamental consideration for liquid biopsy via protein detection is that really useful protein biomarkers may as a rule turn out to be present at very low levels in blood. This is so because proteins that are present at high concentrations in blood from normal individuals are unlikely to be noticeably increased in patients with a limited tumor burden early in the disease or at early recurrence. Greater sensitivity of assays for biomarker proteins that are normally present at low or undetectable levels in blood may translate to earlier detection of disease processes. A case in point outside the sphere of malignant disease is the cardiac troponin isoforms T and I. These structural proteins, specifically expressed in heart muscle, are released to the circulation in ischemic damage of the heart due to myocardial infarction. As sensitivity of troponin assays has gradually improved, the diagnostic indications have expanded to also include stable coronary artery disease, where only low levels of troponins may be present in blood [51].

### 3.1 Affinity‐based protein detection

At present, affinity‐based protein assays, mostly using antibodies as target‐binding reagents, seem to offer the greatest detection sensitivity for protein assays—that is they regularly yield the lowest limits of detection [52]. Such assays also tend to be relatively inexpensive with fast turn‐around times, and they will be in focus here. Nonetheless, steadily improving performance is seen also for assays involving mass spectrometry alone or in combination with affinity reagents, and over time such assay may come to challenge purely affinity‐based detection [53].

We have previously discussed some of the preconditions for sensitive protein detection [54], and we will cover some further aspects here. A first observation is that even the best current protein assays have limited sensitivity, something that restricts opportunities to detect malignancy via protein analysis. Table 1 illustrates the detection sensitivities of some current protein assays to be further discussed below. It should be noted that assay sensitivity also depends on what assay precision is required, and if the assays need to yield results within a short time span. We have chosen to present analytical characteristics for assays detecting interleukin 6 (IL‐6), since IL‐6 is typically present in very low concentrations in the blood (low picograms per mL) [55]. The modest detection sensitivity of even the best current protein assays contrasts with the situation for detection of nucleic acid sequences, where single‐molecule sensitivity is a matter of routine. For example, detection thresholds in routine HIV tests via the two copies of their RNA genome per virus particle are an impressive 50 or even only 20 viral particles per ml of blood [56]. By contrast, at the cutoff for what is considered a high‐sensitivity assays for troponin I one ml of plasma contains around 100 million troponin molecules (around one picogram per mL) [57], a surprisingly high number, although the limit of detection is a few orders of magnitude lower.

**Table 1:** Analytical characteristics of some current protein assays detecting interleukin 6 (IL‐6).

Test	Manufacturer	Type of assay	Assay range (pg·mL^−1^)	Assay range (pm)	Sample volume (µL)	No molecules in sample volume at LLOQ	Multiplex?
IL‐6 Human ProQuantum Immunoassay Kit	Thermo Fisher Scientific	Proximity ligation assay	0.064–10 000	0.003–480	0.5	1000	No
IL‐6 in Immune Response Panel	Olink	Proximity extension assay	0.03–3900	0.001–190	1	1000	Yes, 92‐plex panel
Simoa™ IL‐6 Advantage Kit	Quanterix	Single‐molecule detection assay	0.010–120	0.0005–5.7	20	6000	No
IL‐6 in Simoa™ CorPlex™ Human Cytokine Panel 1	Quanterix	Single‐molecule detection assay	0.148–1200	0.007–57	10	42 000	Yes, 10‐plex panel
Elecsys^®^ IL‐6	Roche	Electrochemiluminescence, sandwich assay	1.5–5000	0.07–240	30	1 300 000	No

### 3.2 Sandwich and proximity assays

**Fig. 2:**
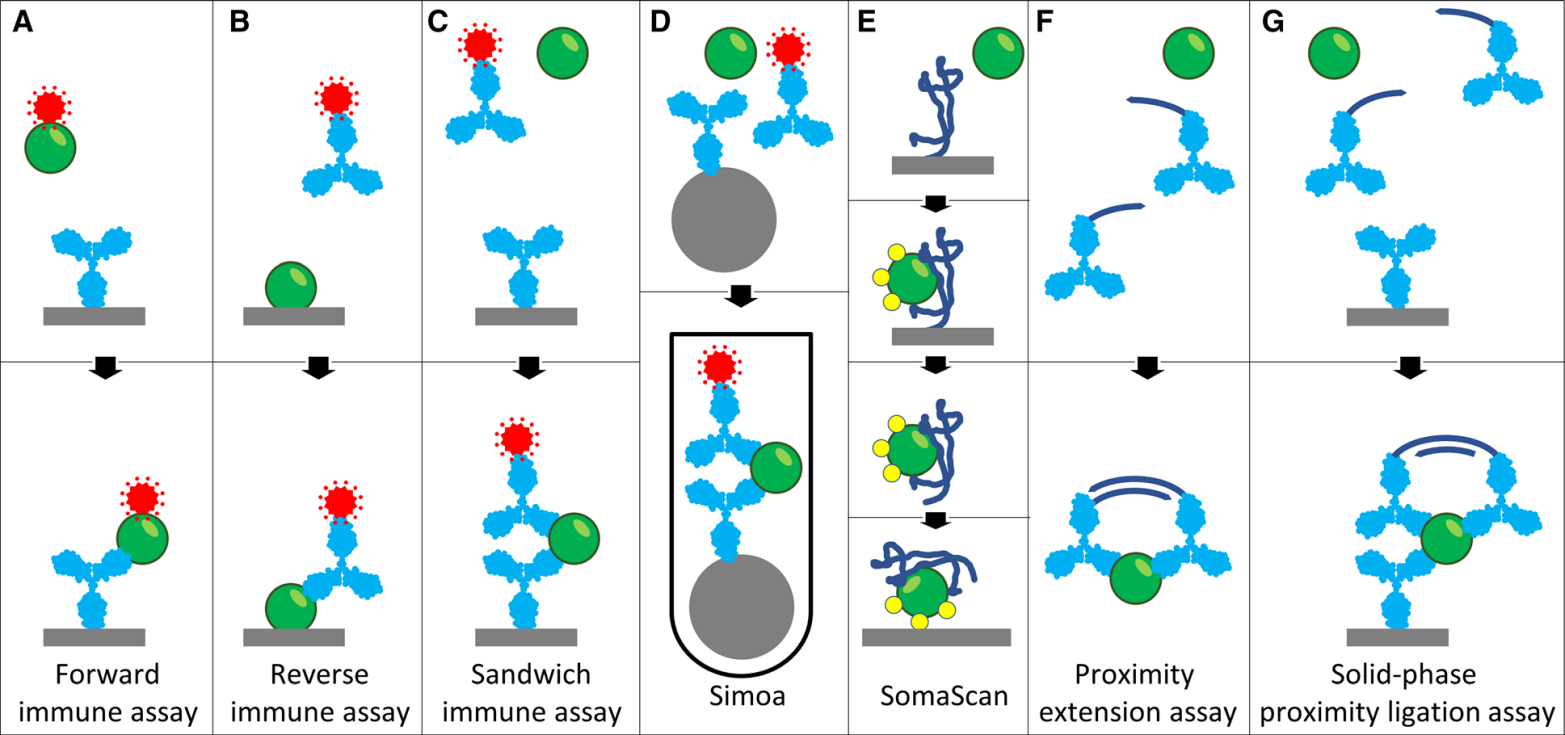
Some formats for affinity‐based protein assays. (A) In forward immune assays, immobilized antibodies (light blue) capture target proteins (green) from samples where all proteins have been labeled with detectable moieties (red). (B) In reverse immune assays, samples are immobilized on solid phases for detection by labeled antibodies. (C) Sandwich immune assays employ pairs of antibodies, one immobilized on a solid support to capture target proteins from solution, and one labeled for detection. (D) The Simoa assay is a variant of the sandwich assays where the particle used as a solid support is confined to a compartment so small that even a single bound detection antibody can elicit a detectable signal, well above any nonspecific signals from media. (E) The SomaScan technique uses immobilized, chemically modified DNA strands (dark blue) as affinity reagents. Upon capture of target proteins and washes, the bound proteins are biotinylated (yellow). Next, the DNA strands and any captured and biotinylated proteins are released for renewed capture on another solid support via the added biotin residues. Finally, the secondarily immobilized probe DNA strands are identified and quantified as a measure of detected proteins. (F) In proximity extension assays pairs of antibodies with conjugated, short DNA strands (dark blue) are incubated with sample, followed by dilution of the reaction and enzymatic extension of DNA strands that remain in proximity by virtue of having bound the same target protein molecule. Finally, the extension products are amplified and recorded by real‐time PCR or DNA sequencing. (G) Solid‐phase proximity ligation assays use trios of target‐specific antibodies, one immobilized for target capture, and two labeled with DNA strands. Oligonucleotides on pairs of antibodies brought in proximity by binding the target protein and remaining after washes are joined by enzymatic DNA ligation, giving rise to reporter DNA strands that can be amplified and recorded as a measure of detected target proteins.

Most commonly, current assays for sensitive protein detection in solution‐phase rely on classical sandwich immune reactions in one form or another [58] (Fig. 2). In these assays, an immobilized antibody captures the target protein from solution, such that it in turn can be bound by a second, labeled antibody, followed by washes and detection of any labeled antibodies that remain indirectly bound to the solid support via the target protein. In contrast to reverse and forward immune assay formats relying of binding by single affinity reagents, sandwich immune assays require pairs of antibodies to recognize the target protein, thereby enhancing specificity.

So‐called proximity ligation or proximity extension assays, originating in our laboratory [59], use a slightly different approach. Just like in sandwich immune assays, each protein must be recognized by two antibodies for proper detection. In proximity assays, each of the two antibodies is conjugated to one of two different DNA oligonucleotides, and the reagents are incubated with the samples in solution. Next, the proximity reactions undergo a dilution step, which takes the place of the washes that are performed in the standard sandwich immune assays. Oligonucleotides on pairs of antibodies that remain in proximity by virtue of having bound the same protein molecule can then undergo DNA ligation (proximity ligation assay) or DNA polymerization (proximity extension assay). The effect of the ligation or polymerization reactions is to create amplifiable reporter DNA strands for sensitive readout via, for example, real‐time PCR or next‐generation sequencing, and the assays can be performed in high multiplex. By constructing the assays so that only proper pairs of antibodies can yield detection signals but no other combination of antibodies, detection of many different proteins in parallel is possible without eroding detection specificity by reactions of noncognate pairs. Proximity assays are among the most highly sensitive protein assays with lower limits of detection in the subpicogram per mL range despite analyzing only 1 µL of sample, but still, typically tens of thousands of target protein molecules must be present in a sample for reliable detection over background in proximity extension assays.

### 3.3 What is limiting detection sensitivity in protein assays?

In principle, it is very simple to see what influences detection sensitivity in affinity‐based protein detection assays. On the one hand, as many as possible of the target molecules in the sample need to be bound by detection reagents in order for them to be detected, and on the other hand, nonspecific detection signals must be kept to a minimum to avoid background that obscures detection of low levels of target proteins. In practice, things get more complicated, and attempts to maximize the efficiency of detection regularly comes at the price of an increased assay background, counteracting detection sensitivity. Accordingly, in protein assays only a proportion of the target proteins in a sample give rise to detection signals, and nonspecific signals limit detection sensitivity. Naturally, efficient detection depends on the quality of the antibodies or other affinity reagents used in the assays, and a myriad other properties of the assay setup. Prominent among these are several factors resulting in target‐independent detection signals in the assays.

### 3.4 Background issues

**Fig. 3:**
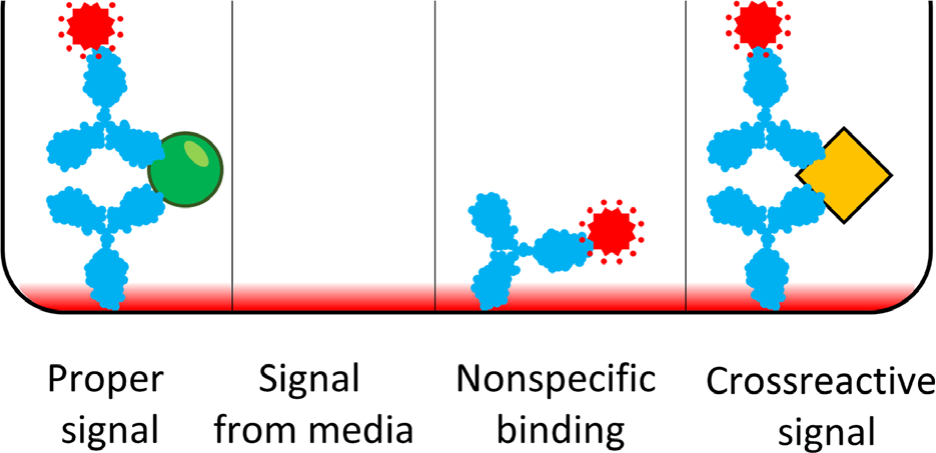
Specific and nonspecific signals in sandwich immune assays. Successful immune reactions give rise to detection signals (red), but contributions to the recorded signal can also arise due to nonspecific signal that may be difficult to distinguish from the correct signals. Such background noise may arise from solutions, reaction vessels, and detection instruments that may exhibit fluorescence or absorbance, etc., that cannot be distinguished from the true signal. Any labeled antibodies that have failed to bind target proteins but that remain after washes due to nonspecific binding will also give rise to background signals. Finally, cross‐reactive binding by the affinity reagents to molecules other than the intended target molecules (yellow) will also contribute to nonspecific signals and thus limit detection signals.

Regarding the nonspecific background below which protein detection is no longer possible, this is the sum of a number of contributing factors (Fig. 3). First, the readout system used may contribute background due to, for example, optical absorbance or fluorescence of solutions and disposables for assays with readout via absorbance or fluorescence, respectively. This type of nonspecific signals obviously does not appear when bound antibodies are detected via attached DNA strands as long as contaminating amplicons are successfully avoided. The Erenna Single Molecule Counting Technology developed by the company Singulex [60] and the Simoa of Quanterix [61] also avoid this source of nonspecific background by confining detection reactions to volume elements so small that only properly labeled detection antibodies can produce enough signals to be recorded, and where even a single labeled antibody suffices for detection. In this sense, these are indeed single‐molecule detection assay as claimed, but this by no mean implies that every single target molecule in the sample may be recorded. A more typical detection threshold in these assays is thousands or tens of thousands of target molecules per sample.

A second source of background is nonspecific binding of detection reagents despite washes. This important contribution to baseline signals is countered by using limiting amounts of detection reagents, balancing the reduction in detection efficiency and by blocking nonspecific binding to surfaces as best one can, but this type of nonspecific signals is difficult to avoid altogether.

One further important contribution to background in protein assays is due to cross‐reactive binding by affinity reagents to molecules in the sample other than the intended target protein molecules. In a typical cytokine assay, the target protein may represent one ten billionth of all the protein molecules present in the sample [48], providing ample opportunities for cross‐reactions with other far more prevalent proteins. The risk for cross‐reactive detection of irrelevant molecules is obviously even greater when there is a need to distinguish between closely similar proteoforms. Here, affinity reagents must be targeted against determinants that distinguish protein variants of interest from any related but incorrect ones.

As already mentioned, this risk for cross‐reactive detection can be significantly reduced if proper detection depends on target binding not only by a single but by pairs of antibodies in sandwich‐type assays [58]. While antibodies can typically bind with some affinity to many proteins besides the ones they were raised against, the risk that a cross‐reactive target would be shared by both antibodies in a sandwich assay is relatively low, this in contrast to assays where binding by a single reagent suffices to elicit detection signals and any act of binding can give rise to detection signals.

A recent, interesting alternative protein assay design, which in fact does depend on target binding by single affinity reagents, is the technology developed by the company SomaLogic with their DNA‐based affinity reagents—so‐called slow off‐rate aptamers or somamers [62]. Their highly multiplexed SomaScan protein assays are constructed so that target proteins must remain bound by their somamers during extended incubations in order to give rise to detection signal. The long washes successfully limit detection to that minority of molecules for which the reagents have been selected to bind with very high affinity, in particular low off‐rate. Also, sandwich assays with their extended washes typically require that proteins remain bound by both the capture and the detection antibodies for some considerable time, thus avoiding detection due to weak cross‐reactive binding.

In proximity extension reactions, by contrast, the oligonucleotide‐conjugated antibody pairs only need to remain bound to their target for a short while upon dilution before the reporter DNA strands form in a DNA polymerization reaction [59, 63]. Despite this opportunity for contribution by antibodies binding with faster off‐rates, the specificity of detection in these assays tends to be very good.

In a series of papers, our laboratory has described sensitive proximity assays where proper protein detection requires coincident target binding by three separate antibodies [59, 64, 65, 66]. These various approaches all serve to enhance detection sensitivity by further reducing risks of cross‐reactive detection of irrelevant protein molecules. The assays also minimize risks of background due to nonspecific adsorption of detection reagents, since no single reagent is capable of giving rise to detectable reaction products, only combinations of them, offering sensitive and specific detection reactions.

All in all, recent years have seen a number of new affinity‐based test architectures for protein assays, some maintaining both high multiplexing and good sensitivity. Nonetheless, there are plenty of opportunities for further improvements of assay techniques as we are currently nowhere near the theoretical limit of truly single‐molecule detection of target proteins in assays for proteins in blood. Novel detection techniques offering greatly enhanced detection sensitivities may well result in a new generation of assays for previously undetectable predictive protein biomarkers in cancer for early detection or to monitor the clinical course of cancer patients.

## 4 Conclusions and future directions

Plasma proteins were the first targets for what is now referred to as liquid biopsy, only in recent years joined by a number of other classes of molecules and circulating cells. As discussed herein, cancer diagnostics via protein assays got off to a slow start, but there is an increasingly well‐resolved understanding of protein complements of cell types throughout the body, with some proteins potentially representing attractive targets for diagnostics. Protein assay technology is also developing to allow for improved performance. Accordingly, there is great hope that protein assays will become available for successful screening for malignancy or evaluation of responses to therapy in most common malignancies. The importance of improved cancer diagnostics via protein assays could prove vast. Assays capable of detecting cancer at early stages, allowing malignant processes to be interrupted before metastatic spread, can prove life saving for the individuals concerned, and they can impact society as a whole by reducing healthcare costs, preserving active years, and building new industries of diagnostic testing.

The statistical requirements for validating a burst of new potential protein markers mean that we will need ever larger patient sample collections, accompanied by excellent clinical data. Validation of proposed biomarkers for clinical use has proven a major stumbling block so far, and this need will only continue to grow. The challenge to achieve statistical significance will be further augmented as future protein markers are likely not to be used individually but in panels that only jointly achieve the required diagnostic efficiency and predictive value.

To find and validate new protein biomarkers, there is also a need to build biobanks with numerous consecutive samples collected over time from each of large numbers of individuals. Such consecutive samples can be helpful to define the normal concentration ranges for individuals, as these ranges frequently differ widely among different people because of both genetic [67, 68] and environmental factors [69]. Consecutive testing has been shown to considerably improve the diagnostic power of the CA‐125 cancer marker [70]. Collections of longitudinal samples from large population‐based cohorts will also have the effect of increasing the probability that samples are available for testing from those individuals who go on to develop a disease, and that samples may have been collected at timepoints in the course of the disease when an early, helpful diagnosis could have been made, before any onset of symptoms. Such samples will be crucial to evaluate predictive biomarkers in retrospective patient materials.

It turns out that sets of proteins can be advantageously measured in dried blood spots that individuals can collect themselves by pricking a finger and sending the sample by mail, with very low transaction costs for storage and collection [71, 72]. This opportunity further emphasizes the need for highly sensitive assays because of the limited sample volumes available in blood spots. Nonetheless, some applications may still require larger sample volumes that contain more copies of rare molecules of interest, in order to achieve the requisite molar sensitivity and analytical precision.

All in all, finding plasma protein cancer biomarkers of the future will require a confluence of biological and medical insights greatly improved molecular assay platforms, and the logistics of vast amounts of patient samples. Ultimately, successfully developed assays may help realize the hope for effective population screening for early signs of malignant disease to enable curative surgery and in anticipation of steadily improving medical therapies.

## Conflict of interest

Ulf Landegren is a co‐founder and shareholder of Olink Proteomics and Navinci Diagnostics, having rights to the proximity extension assay (PEA) and proximity ligation assay (PLA) technologies.

## Author contribution

UL and MH wrote the manuscript.
